# A case report and diagnostic-therapeutic analysis of *de novo* bilateral papillary thyroid carcinoma complicated with stage I a diffuse large B-cell lymphoma (ABC subtype, double-expressor)

**DOI:** 10.3389/fonc.2026.1921091

**Published:** 2026-09-21

**Authors:** Lijing Wang, Yang Zhou, Long Liu, Jinfeng Tian, Zhengyao Zhang, Ziyi Shao, Jingfei Shi, Shumei Liu, Chao Cui

**Affiliations:** 1 Department of Hematology, Qilu Hospital of Shandong University Dezhou Hospital, Dezhou, China; 2 Department of Hematology, The Affiliated Taian City Central Hospital of Qingdao University, Taian, Shandong, China; 3 Medical School, Shandong Xiehe University, Jinan, Shandong, China; 4 Department of Clinical and Basic Medicine, Shandong First Medical University, Jinan, Shandong, China; 5 Scientific Research Department, Qilu Hospital of Shandong University Dezhou Hospital, Dezhou, China

**Keywords:** case report, diffuse large B-cell lymphoma, double primary malignant tumors, immunochemotherapy, neutropenia, papillary thyroid carcinoma, polatuzumab vedotin

## Abstract

Diffuse large B-cell lymphoma (DLBCL) is the most common aggressive non-Hodgkin lymphoma in adults, and papillary thyroid carcinoma (PTC) is the most prevalent type of thyroid malignant tumor. Chronic systemic immune dysregulation, constitutive overactivation of the PI3K/AKT/mTOR and NF-κB signaling axes, reshaped immunosuppressive tissue microenvironment and aberrant B-cell clonal expansion serve as core shared molecular and immunological mechanisms that synergistically predispose patients to synchronous tumorigenesis of papillary thyroid carcinoma and ABC-type double-expressing diffuse large B-cell lymphoma. Both are clinically high-incidence malignancies; however, cases with synchronous diagnosis of *de novo* double primary DLBCL and PTC are extremely rare in clinical practice. Due to potential symptom overlap or lesion location correlation between the two diseases, misdiagnosis and missed diagnosis are likely to occur. Treatment regimens need to take into account the pathophysiological characteristics, clinical staging of both tumors, and the patient’s individual tolerance, leading to high difficulty in clinical decision-making. This article reports a 49-year-old female patient with *de novo* double primary bilateral PTC (with right cervical lymph node metastasis, metastasis rate 4/6) and stage I A DLBCL (not otherwise specified, activated B-cell-like subtype, double-expressor). The diagnosis was confirmed by preoperative imaging evaluation, puncture biopsy pathology, as well as postoperative specimen pathology, immunohistochemistry, and fluorescence *in situ* hybridization (FISH) detection. The patient received radical thyroidectomy combined with polatuzumab vedotin (Pola)-anti-CD20 monoclonal antibody-CHP regimen for immunochemotherapy. Grade III neutropenia occurred during treatment, which returned to normal after symptomatic treatment and secondary prevention, with good tolerance in subsequent treatment. After 6 cycles of immunochemotherapy, DLBCL achieved complete remission (CR), and the patient recovered well after thyroidectomy, completing planned iodine-131 therapy postoperatively. As of October 2025, the total follow-up duration was 6 months, with no signs of tumor recurrence noted to date; nevertheless, longer-term continuous surveillance is still required to definitively exclude late relapse risk, given the aggressive nature of double-expressor DLBCL. Meanwhile, combined with the diagnosis and treatment process of this case and relevant literature, we discuss the clinical characteristics, diagnostic key points, and individualized treatment strategies of double primary DLBCL and PTC, aiming to provide a reference for the standardized diagnosis and treatment of similar rare clinical cases.

## 1 Introduction

Diffuse large B-cell lymphoma (DLBCL) is the most common aggressive adult non-Hodgkin lymphoma (30%-40% incidence). It features strong pathological heterogeneity; the activated B-cell-like (ABC) subtype bears inferior prognosis driven by aberrant signaling pathways and demands personalized therapy (1). Papillary thyroid carcinoma (PTC) accounts for over 80% of thyroid malignancies. Though usually indolent with excellent long-term survival after standard therapy, clinicians must monitor its cervical lymph node metastasis and postoperative recurrence risks (2). Double primary malignancies are defined by independent tissue origins and distinct pathology, with metastatic lesions excluded. Synchronous *de novo* DLBCL plus PTC is extremely rarely reported, and unified diagnostic and therapeutic guidelines for this comorbidity are lacking (3).

Clinically, both diseases cause cervical lymphadenopathy, easily triggering misdiagnosis: thyroid patients’ cervical nodes are frequently judged as PTC metastasis, while concurrent primary lymphoma lesions are ignored. Comprehensive multi-modal examinations are required for definitive diagnosis. At the molecular and cellular level, multiple overlapping oncogenic aberrations serve as common pathogenic drivers of synchronous tumorigenesis: concomitant overactivation of the PI3K/AKT/mTOR and NF-κB signaling axes, chronic systemic immune microenvironment disorder, germline susceptibility variants, and persistent chronic inflammatory stimulation collectively impair host immune surveillance function (4, 5). Immune dysfunction is hypothesized as a shared predisposing factor: DLBCL arises from B-cell signaling defects and weakened immune surveillance, whereas PTC is linked to BRAF/RET alterations and local thyroid immune microenvironment disorders. Their overlapping immune network abnormalities may promote synchronous tumorigenesis, yet specific molecular mechanisms remain to be validated.

Treatment requires balancing local and systemic management. Radical surgery plus optional postoperative iodine-131 therapy is the mainstay for PTC; immunochemotherapy serves as the core systemic treatment for DLBCL, tailored to staging, subtype and prognostic markers. However, optimal treatment sequence, regimen matching and adverse event management lack high-level evidence, bringing great challenges to clinical decision-making. Given the rarity and therapeutic complexity of this double primary tumor, we herein report a case of synchronous bilateral PTC combined with stage I A ABC-type double-expressor DLBCL. We summarize its diagnostic workflow, therapeutic strategy and follow-up results, to offer clinical references for standardized management of similar rare double-primary tumor cases.

## 2 Case presentation

### 2.1 General information

A 49-year-old female patient presented to the Breast and Thyroid Surgery Outpatient Clinic of our hospital on November 23, 2024, due to “discovery of a cervical mass for 1 week”. She had no symptoms such as fever, night sweats, unexplained weight loss, dysphagia, hoarseness, dyspnea, breast distension or nipple discharge. She had no history of chronic underlying diseases, family history of malignant tumors, radiotherapy, chemotherapy, or drug allergies. After admission, basic laboratory examinations including routine blood tests, liver and kidney function, and electrolytes were completed, and all results were unremarkable (**Supplementary Table 1**).

### 2.2 Preliminary diagnostic examinations

#### 2.2.1 Thyroid and cervical lymph node ultrasonography

Thyroid ultrasonography revealed a solid nodule in the right lobe, measuring approximately 0.8 cm × 0.9 cm, with an aspect ratio > 1, ill-defined margins, heterogeneous internal echo, and microcalcifications, classified as C-TIRADS category 4c. Scattered solid nodules (C-TIRADS category 4a), cystic nodules, and complex cystic-solid nodules (C-TIRADS category 3) were observed in the remaining thyroid parenchyma, without obvious capsule invasion signs (**Figure 1A**). Cervical lymph node ultrasonography showed multiple abnormally enlarged lymph nodes in the right neck, with the largest dimension of approximately 0.9 cm × 0.5 cm, presenting hypoechoic internal echo and indistinct cortex-medulla differentiation, suggestive of metastatic or proliferative lesions (**Figure 1B**). An abnormally enlarged lymph node was detected in the left neck level IV, measuring about 2.0 cm × 1.1 cm, with loss of cortex-medulla differentiation, indicating possible proliferative changes and cannot rule out lymphoma involvement; further pathological biopsy was recommended to confirm the nature (**Figure 1C**).

**Figure 1:**
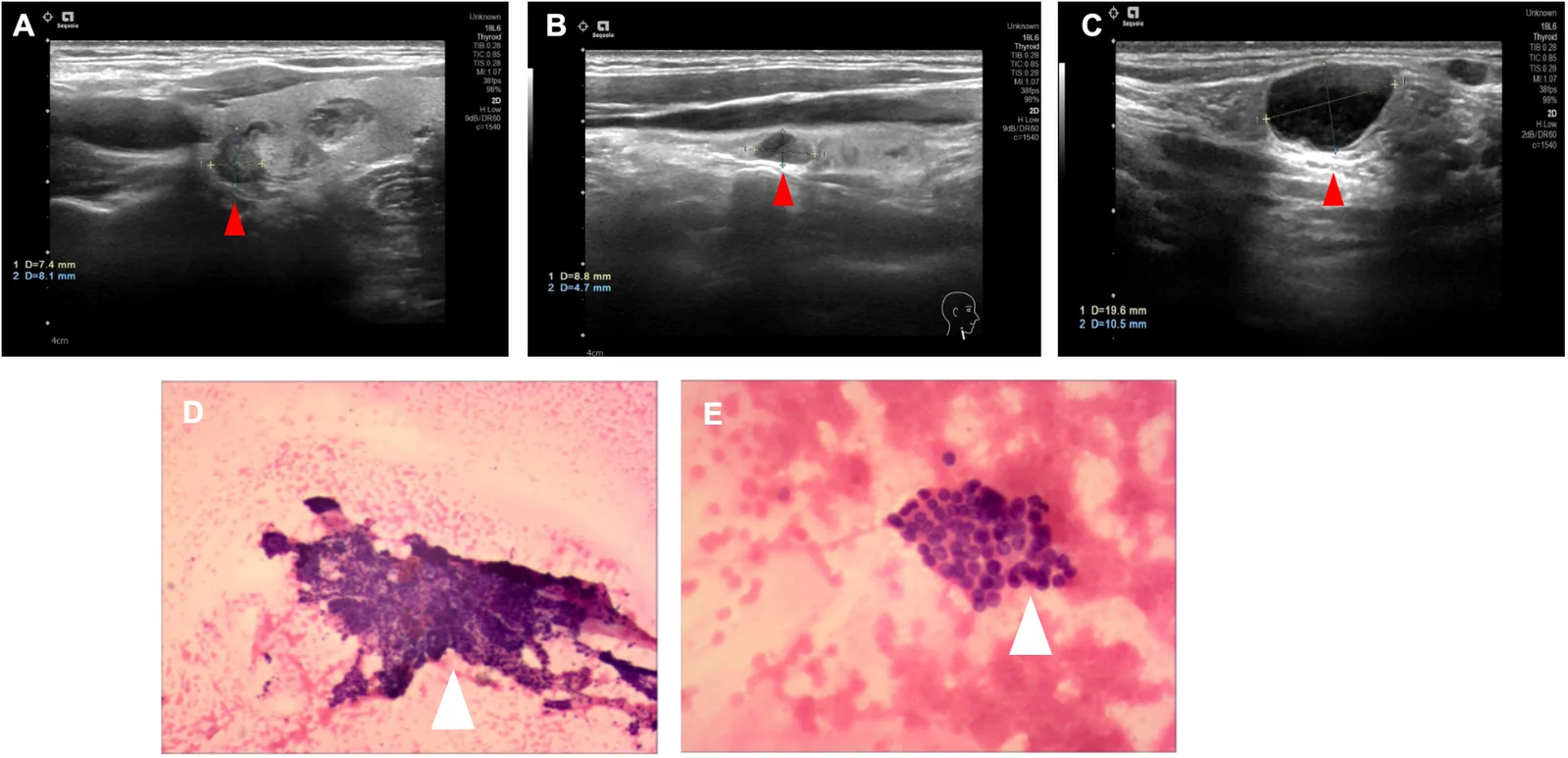
Ultrasonographic findings of cervical lymph nodes and preoperative thyroid fine-needle aspiration biopsy pathology results. **(A)** Ultrasonographic features of a C-TIRADS category 4c nodule in the right thyroid lobe (arrow indicates aspect ratio > 1, solid nodule with hypoechogenicity, irregular margins, and punctate hyperechogenicity within the nodule). **(B)** Ultrasonographic features of a lymph node in the right neck level IV (arrow indicates hypoechogenicity and ill-defined hilum, suggesting suspected papillary thyroid carcinoma metastasis). **(C)** Ultrasonographic features of a lymph node in the left neck level IV (arrow indicates hypoechogenicity, ill-defined hilum, and irregular morphology, suggesting suspected lymphoma). **(D)** Preoperative thyroid fine-needle aspiration biopsy pathology: consistent with papillary thyroid carcinoma (HE × 100). **(E)** Preoperative thyroid fine-needle aspiration biopsy pathology: consistent with papillary thyroid carcinoma (HE × 200).

#### 2.2.2 Preoperative thyroid fine-needle aspiration biopsy pathology

Pathological results of bilateral thyroid fine-needle aspiration biopsy showed that both bilateral thyroid tissues were consistent with the diagnosis of papillary thyroid carcinoma (**Figures 1D, E**). Considering the abnormal cervical lymph node findings indicated by ultrasonography, surgical treatment was planned to clarify the extent of thyroid tumor and the nature of cervical lymph node lesions. The proposed surgical procedure was total thyroidectomy plus bilateral central neck lymph node dissection, bilateral level IV cervical lymph node biopsy, radical dissection of right neck level II, III, IV, and V lymph nodes, and bilateral recurrent laryngeal nerve exploration.

### 2.3 Surgical treatment

The patient was diagnosed with bilateral PTC by preoperative biopsy, with definite abnormal signs of cervical lymph nodes. She had surgical indications and no contraindications to surgery. On December 6, 2024, she underwent total thyroidectomy plus bilateral central neck lymph node dissection, bilateral level IV cervical lymph node biopsy, and radical dissection of right neck level II, III, IV, and V lymph nodes under general anesthesia. The operation was performed smoothly, and no complications such as recurrent laryngeal nerve injury or massive hemorrhage occurred intraoperatively.

### 2.4 Postoperative examinations

#### 2.4.1 Postoperative pathological examination

To clarify the specific nature of thyroid lesions and cervical lymph node lesions, routine pathological examinations were performed on the surgically resected specimens. The results were as follows: ① Bilateral thyroid lobes and isthmus: both were confirmed as papillary thyroid carcinoma (**Figures 2A, B**); ② Right neck level IV lymph nodes (6 nodes): metastatic carcinoma was detected in 4 nodes, with morphology consistent with papillary thyroid carcinoma metastasis (**Figure 2C**); ③ Left neck level IV lymph nodes: diffuse proliferation of atypical lymphocytes was observed under the microscope, with morphology consistent with DLBCL (**Figure 2D**).

**Figure 2:**
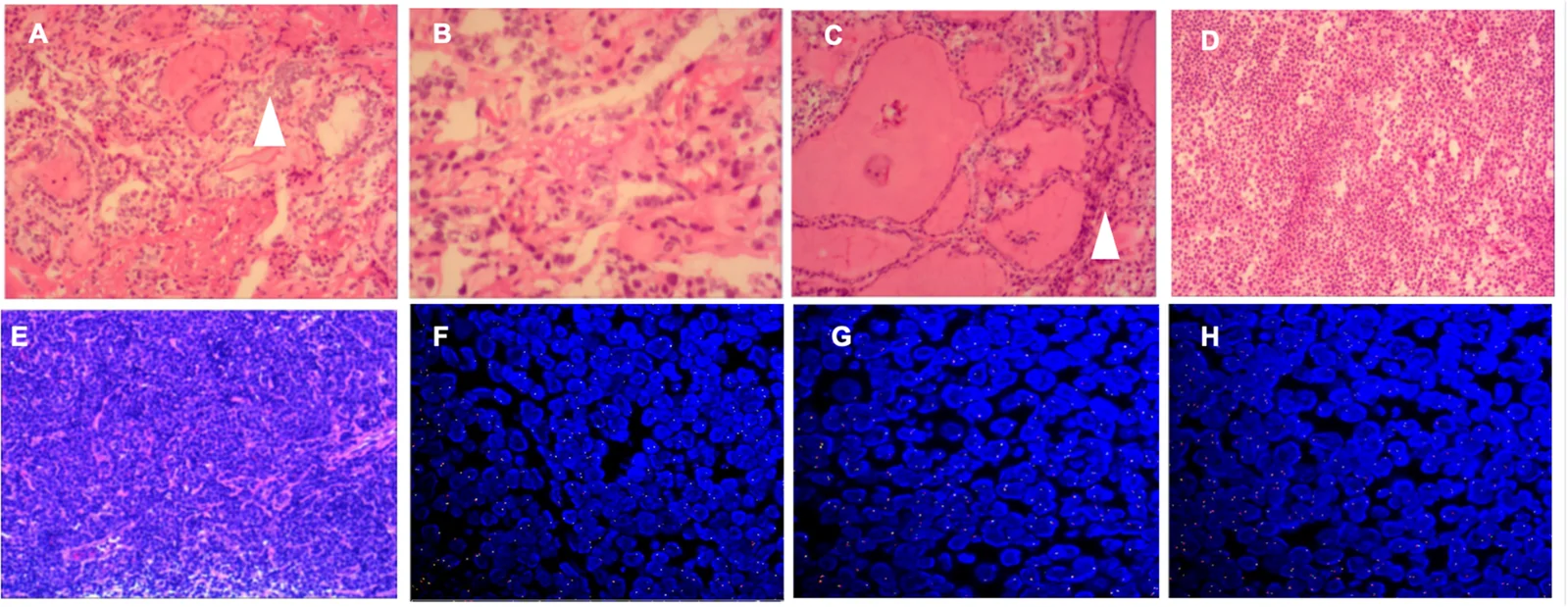
Postoperative HE staining, ISH and FISH results of thyroid lesions and cervical lymph node specimens. **(A)** Postoperative thyroid tissue pathology: papillary thyroid carcinoma (HE × 100). **(B)** Postoperative thyroid tissue pathology: papillary thyroid carcinoma (HE × 200). **(C)** Postoperative pathology of right neck level IV lymph nodes: papillary thyroid carcinoma metastasis detected (HE × 100). **(D)** Postoperative pathology of left neck level IV lymph nodes: diffuse proliferation of atypical lymphocytes observed (HE × 100). **(E)** Left cervical lymph node: EBER *in situ* hybridization (EBER-negative, no nuclear signal). **(F)** BCL2 FISH detection result of left cervical lymph nodes (no abnormal amplification). **(G)** BCL6 FISH detection result of left cervical lymph nodes (no abnormal amplification). **(H)** MYC FISH detection result of left cervical lymph nodes (no abnormal amplification).

#### 2.4.2 *In situ* hybridization and fluorescence *in situ* hybridization detection

Further ISH and FISH assays were performed on the left neck level IV lymph node specimen. ISH detected Epstein-Barr virus (EBV)-encoded RNA (EBER) (-) (**Figure 2E**). Immunohistochemical staining further confirmed the dual-expression profile: MYC protein expression was positive in approximately 45% of tumor cells, and BCL2 protein was diffusely positive in approximately 80% of tumor cells, meeting the diagnostic criteria for MYC/BCL2 double-expression DLBCL. FISH revealed no abnormal amplification of BCL2, BCL6, or MYC genes, which was consistent with the diagnostic criteria for DLBCL, ABC subtype (**Figures 2F–H**). For bilateral papillary thyroid carcinoma lesions, no additional molecular genetic testing (including BRAF V600E, TERT promoter, and RAS mutation detection) was performed in this routine pathological examination, which represents a limitation of the present case. Finally, the patient was diagnosed with DLBCL, not otherwise specified (NOS), ABC subtype, double-expressor.

#### 2.4.3 Laboratory examinations and bone marrow evaluation

Postoperative re-examinations including routine blood tests, liver and kidney function, and electrolytes showed all results remained normal (**Supplementary Table 2**). Tumor marker detection (**Figure 3A**) revealed: lactate dehydrogenase (LDH) 215 U/L (reference range: 120–250 U/L) and β2-microglobulin (β2-MG) 1.8 mg/L (reference range: 1.0-3.0 mg/L), both within normal limits. The Eastern Cooperative Oncology Group (ECOG) performance status score was 2. To rule out bone marrow infiltration by DLBCL, bone marrow cytomorphological examination and bone marrow biopsy were performed. The results showed no abnormal lymphocytes in bone marrow cytology (**Figure 3B**), and no signs of lymphoma cell infiltration were found in bone marrow pathological sections (**Figure 3C**), confirming the exclusion of bone marrow involvement.

**Figure 3:**
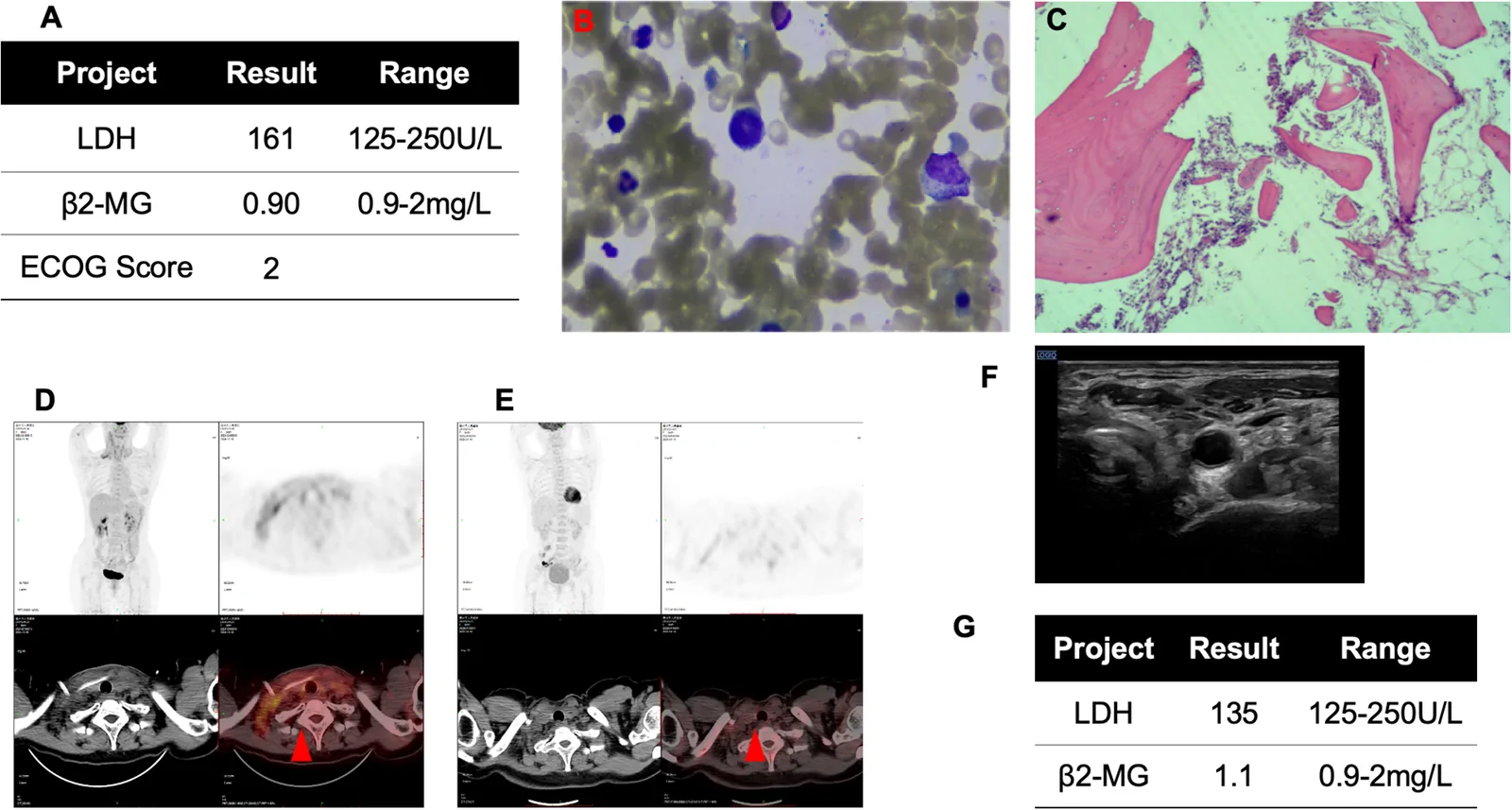
Postoperative laboratory examinations, bone marrow evaluation, PET/CT, and post-treatment follow-up examination results. **(A)** Postoperative LDH, β2-MG detection results (both normal) and ECOG performance status score (2 points). **(B)** Bone marrow cytomorphological examination: no abnormal lymphocytes observed (Wright’s stain × 400). **(C)** Bone marrow pathological biopsy: no lymphoma cell infiltration (HE × 100). **(D)** Postoperative pre-treatment PET/CT: After total thyroidectomy, catheter shadow was seen in the surgical area with diffuse increased glucose metabolism, SUVmax: 6.8 (arrow indicates diffuse increased glucose metabolism, SUVmax: 6.8, considered postoperative changes). **(E)** PET/CT after 6 cycles of immunochemotherapy: No abnormal metabolism was found in the original DLBCL lesions and thyroid cancer surgical area, indicating complete remission of DLBCL (arrow indicates no abnormal metabolic activity). **(F)** Cervical lymph node ultrasonography during follow-up after chemotherapy completion: no abnormally enlarged lymph nodes. **(G)** LDH and β2-MG detection results during follow-up (both remained normal).

#### 2.4.4 Postoperative positron emission tomography/computed tomography evaluation

The patient underwent whole-body PET/CT examination on December 16, 2024 (**Figure 3D**). The results were as follows: ① Thyroid cancer-related lesions: Post-thyroidectomy, right cervical soft tissue edema with hypermetabolism was noted, considered postoperative changes; ② Lymphoma-related lesions: No obvious abnormally increased metabolic foci were found in other parts of the body (including lymph nodes in regions other than the left neck, thoracoabdominal organs, skeletal system, etc.), with no evidence of distant invasion by DLBCL; ③ Other abnormal signs: Multiple small nodules without metabolic activity in both lungs were considered benign lesions; mild thickening of the left adrenal gland with no abnormal metabolic activity was suggestive of possible proliferative changes.

### 2.5 Final postoperative diagnosis

Combining preoperative examination results, pathological diagnosis of surgically resected specimens, postoperative systemic evaluation, and auxiliary test findings, the final diagnosis was made as follows: ① Bilateral papillary thyroid carcinoma (right cervical lymph node metastasis, metastasis rate 4/6); ② DLBCL (not otherwise specified, ABC subtype, double-expressor), Ann Arbor stage I A (left neck level IV lymph node involvement), age-adjusted International Prognostic Index (aaIPI) score of 1 (intermediate-low risk group). The patient recovered well postoperatively without surgery-related complications such as recurrent laryngeal nerve injury or hypoparathyroidism, and planned to complete postoperative iodine-131 therapy for thyroid carcinoma after physical status recovery.

## 3 Treatment methods

### 3.1 Immunochemotherapy regimen for DLBCL

One month after surgery (January 2025), the patient’s physical status recovered well with an improved ECOG performance status score of 1, and systemic treatment for DLBCL was initiated. Based on the patient’s DLBCL clinical stage (stage I A), pathological subtype (ABC subtype, double-expressor), and aaIPI score (1 point, intermediate-low risk), with reference to the recommendations in the 2024 edition of the *Chinese Society of Clinical Oncology (CSCO)* Guidelines for the Diagnosis and Treatment of Lymphoma, standard-dose Pola-anti-CD20 monoclonal antibody-CHP immunochemotherapy was administered. Each treatment cycle was 21 days, and 6 cycles of standardized treatment were planned. The Pola-R-CHP regimen replaces vincristine in the classic R-CHOP backbone with polatuzumab vedotin, consisting of rituximab, cyclophosphamide, doxorubicin, prednisone and polatuzumab vedotin. For this patient, postoperative iodine-131 ablation therapy for papillary thyroid carcinoma was arranged to be implemented after completion of all six cycles of immunochemotherapy; iodine-131 treatment and immunochemotherapy were not administered concurrently.

### 3.2 Adverse reaction management

After completion of the first cycle of immunochemotherapy, routine blood re-examination (**Supplementary Table 3**) indicated Grade III neutropenia (neutrophil count 0.8×10^9^/L). The patient had no infection-related symptoms such as fever, cough, or abdominal pain, nor other obvious discomfort. Recombinant human granulocyte colony-stimulating factor was immediately administered for symptomatic leukocytosis treatment, and subsequent routine blood re-examination showed that the neutrophil count recovered to the normal range. To prevent recurrence of myelosuppression in subsequent treatment, pegylated granulocyte colony-stimulating factor 6 mg was routinely administered subcutaneously for secondary prevention after the end of the second cycle of immunochemotherapy. During the subsequent 5 cycles of treatment, no neutropenia recurred, nor were there obvious chemotherapy-related adverse reactions such as nausea, vomiting, alopecia, liver or kidney function impairment, or peripheral neurotoxicity. The patient had good treatment tolerance, completed all cycles of treatment as planned, with no treatment interruption.

## 4 Treatment outcomes

### 4.1 Efficacy evaluation

After completion of 6 cycles of Pola-anti-CD20 monoclonal antibody-CHP immunochemotherapy (April 2025), the patient underwent whole-body PET/CT re-examination to assess DLBCL treatment efficacy and monitor postoperative recovery of thyroid carcinoma. The results were as follows: ① DLBCL efficacy: Compared with preoperative PET/CT images, no obvious abnormally metabolically active lymph nodes or tumor lesions were detected in all parts of the body, and no residual metabolic signs of lymphoma were found in the left cervical surgical area. According to lymphoma efficacy evaluation criteria, DLBCL was assessed as complete remission (CR) (**Figure 3E**); ② Postoperative recovery of thyroid carcinoma: No abnormally metabolically active lesions were observed in the thyroid surgical area, indicating good postoperative recovery (**Figure 3E**); ③ Other follow-up signs: Multiple small nodules without metabolic activity in both lungs and mild thickening of the left adrenal gland were consistent with the previous examination, with no obvious progression, and regular follow-up monitoring was planned.

### 4.2 Follow-up

After completion of DLBCL immunochemotherapy, in accordance with the follow-up guidelines for lymphoma and thyroid carcinoma, the patient underwent re-examinations of cervical lymph node ultrasonography (**Figure 3F**), routine blood tests (**Supplementary Table 4**), LDH, and β2-MG (**Figure 3G**) every 3 months, with simultaneous monitoring of thyroid function and thyroglobulin levels. As of October 2025, the total follow-up duration was 6 months. During the follow-up period, there was no cervical lymphadenopathy; laboratory indicators including routine blood tests, LDH, and β2-MG remained within normal ranges, with no signs of DLBCL recurrence. The patient completed the planned postoperative iodine-131 therapy sequentially after the full 6 cycles of DLBCL immunochemotherapy were fully finished (no overlapping administration with immunochemotherapy), re-examination showed well-controlled thyroid function and normal thyroglobulin levels, with no evidence of thyroid carcinoma recurrence or distant metastasis.

It should be noted that the 6-month follow-up period is relatively short for long-term prognosis assessment of both malignancies, which constitutes a limitation of the present case report. Long-term continuous follow-up will be maintained to monitor long-term disease outcome.

## 5 Discussion

Against the scarcity of unified guidelines for synchronous DLBCL and PTC double primary lesions, we further dissect the potential shared molecular pathogenesis and extract clinical operational experience from this case. At the cellular and molecular level, multiple overlapping core signaling cascades and tumor microenvironment disorders serve as key shared pathogenic drivers. The persistent overactivation of the PI3K/AKT/mTOR axis and constitutive NF-κB pathway represent the most vital common molecular link: in PTC, BRAF V600E and RET fusions constitutively upregulate NF-κB and PI3K/AKT signaling, remodeling the local thyroid immune microenvironment by recruiting immunosuppressive cells, impairing tissue immune surveillance; in ABC-type double-expressing DLBCL, chronic B-cell receptor signaling also sustains NF-κB and PI3K/AKT hyperactivation, driving MYC/BCL2 co-overexpression and intrinsic immune escape of lymphoma cells. Beyond oncogenic signaling, germline genetic susceptibility variants and chronic inflammatory stimulation jointly disrupt systemic immunoregulation, creating a systemic “tumor-prone inflammatory niche” that facilitates the synchronous oncogenic transformation of thyroid follicular epithelial cells and mature B lymphocytes (6).

Clinically, cervical lymph node lesions in PTC patients have a high missed diagnosis risk for concurrent lymphoma. Based on this case, we propose a standardized differential diagnosis workflow for such high-risk populations: ① Imaging evaluation: Thyroid nodules classified as C-TIRADS category 4c indicate a high malignant risk. When cervical lymph nodes present abnormal signs such as irregular morphology and loss of cortex-medulla differentiation, lymphoma must be listed in differential diagnosis rather than simply attributing lesions to PTC metastasis; complete pathological sampling is indispensable for final diagnosis. ② Pathological diagnosis: Thyroid fine-needle aspiration biopsy has inherent limitations for heterogeneous cervical lymph nodes and often fails to rule out combined lymphoma. Surgically excised intact lymph node specimens, combined with immunohistochemistry and FISH molecular detection, constitute the gold standard to distinguish metastatic carcinoma and synchronous primary lymphoma. ③ Systemic evaluation: Combined PET/CT and bone marrow biopsy can complete accurate staging of DLBCL and exclude distant organ infiltration, which provides critical evidence for subsequent treatment decision-making.

This case’s therapeutic sequence and regimen selection provide practical references for managing synchronous PTC and limited-stage ABC double-expressor DLBCL. The treatment of double primary DLBCL and PTC should follow the core principles of “individualized treatment and balancing the diagnostic and therapeutic needs of both tumors”. The regimen should be formulated based on tumor aggressiveness, clinical staging, and the patient’s individual tolerance, with optimized treatment sequence and strategy: ① Thyroid cancer treatment focuses on radical surgical resection. Postoperatively, iodine-131 therapy is supplemented according to lymph node metastasis status and thyroid cancer pathological subtype to achieve precise local tumor control and reduce the risk of local recurrence. ② For DLBCL treatment, individualized regimens should be selected based on clinical staging, pathological subtype, and prognostic index. It is well acknowledged that non-bulky stage IA conventional DLBCL usually receives 3–4 cycles of immunochemotherapy followed by involved-site radiotherapy (ISRT) to shorten treatment duration and lower immunosuppression. However, this patient carried two high-risk pathological features: ABC molecular subtype and MYC/BCL2 double-expressor status. Even at stage IA, early-stage double-expressor ABC-type DLBCL has higher chemoresistance and occult residual lesion risk, and shortened therapy is prone to subsequent disease relapse. Thus, we chose a full 6-cycle Pola-CHP regimen rather than a reduced cycle plus radiotherapy to completely eradicate potential microscopic lymphoma lesions. Our decision to omit consolidative radiotherapy was based on several patient-specific considerations. First, the patient had already undergone total thyroidectomy and comprehensive bilateral cervical lymph node dissection. In this context, the primary rationale for ISRT—organ preservation—was no longer applicable, and the altered postsurgical anatomy increases the complexity of target volume delineation, potentially reducing the precision of radiotherapy while increasing exposure of adjacent normal tissues. Second, administering early postoperative radiotherapy to the neck would likely compound surgical trauma, potentially leading to delayed wound healing, progressive cervical fibrosis, lymphedema, and permanent functional impairment, which could severely affect the patient’s long-term quality of life. Third, as recommended by NCCN guidelines, six cycles of systemic immunochemotherapy without ISRT is an accepted alternative for patients with limited-stage non-bulky DLBCL who are unsuitable for or wish to avoid radiotherapy. Therefore, our strategy of full-course Pola-R-CHP without ISRT appropriately balanced the need for aggressive systemic control of this biologically high-risk lymphoma against the imperative to preserve cervical function and avoid irreversible local damage in the post-thyroidectomy setting. Notably, although the patient presented with intermediate-low aaIPI score and would conventionally qualify for standard R-CHOP therapy, we adopted Pola-R-CHP based on subtype-specific biological characteristics and high-level clinical evidence. The clinical IPI scoring system only reflects macroscopic clinical risk and cannot represent tumor molecular biological behavior. First, ABC-type DLBCL survives depending on persistent NF-κB pathway activation induced by frequent MYD88 or CARD11 mutations, which causes inherent resistance to vincristine contained in traditional R-CHOP. Polatuzumab vedotin is a CD79b-targeted antibody-drug conjugate that kills B-cell lymphoma cells by releasing intracellular MMAE, exerting anti-tumor effects completely independent of the NF-κB signaling status. This mechanism effectively bypasses the drug-resistance defect of ABC-subtype DLBCL and compensates for the insufficient efficacy of standard R-CHOP. Second, the predefined subgroup analysis of the landmark POLARIX trial strongly supports our strategy. In non-GCB/ABC subtype populations, Pola-R-CHP significantly improved progression-free survival compared with R-CHOP, with a HR of 0.38 (95%CI 0.24-0.59), demonstrating dramatic subtype-specific survival benefits regardless of IPI score (7). Third, Pola-R-CHP only replaces vincristine with polatuzumab vedotin while retaining the entire R-CHOP drug backbone. The peripheral neurotoxicity is comparable to conventional R-CHOP, and the main adverse reaction is neutropenia, which can be fully controlled by standardized G-CSF prophylaxis, ensuring reliable safety and treatment compliance. Fourth, individualized comprehensive consideration was essential for this early-stage but biologically high-risk case. Although the patient was stage I A with only one IPI risk factor, double-expressor ABC-type DLBCL carries an extremely aggressive biological nature. Once relapse occurs, it often progresses to advanced-stage refractory lymphoma with very limited salvage treatment options. Therefore, under the premise of good physical tolerance and feasible economic conditions, we performed subtype-oriented therapeutic escalation to achieve deeper complete remission and minimize early recurrence risk. In addition, we would like to clarify the concern regarding immunosuppression aggravated thyroid cancer progression: radical total thyroidectomy and complete cervical lymph node dissection had radically removed all viable thyroid cancer metastatic lesions before lymphoma immunochemotherapy. There was no residual active PTC lesion in the body, so the immunosuppressive effect of prolonged chemotherapy could not cause deterioration or progression of thyroid carcinoma. Furthermore, the immunosuppression induced by immunochemotherapy is predominantly transient and reversible, primarily affecting B-cell lineages and neutrophil counts, with limited long-term impact on T-cell-mediated antitumor immunity, which is less critical for the indolent nature of completely resected differentiated PTC. Delaying radioactive iodine therapy until after the completion of all six cycles of immunochemotherapy is safe and does not compromise the curative opportunity for PTC, given its generally slow progression. For DLBCL patients with ABC subtype, double-expressor phenotype, Ann Arbor stage I A, and intermediate-low risk by aaIPI, current guidelines recommend immunochemotherapy as the core treatment. Among them, the Pola-anti-CD20 monoclonal antibody-CHP regimen can enhance antitumor efficacy through the targeted cytotoxic effect of antibody-drug conjugate, especially showing superior therapeutic advantages in DLBCL patients with ABC subtype. Our patient’s treatment experience further proves that this regimen achieves favorable efficacy in such complicated comorbid patients. The Grade III neutropenia emerging in the first cycle was effectively controlled by symptomatic intervention and preventive long-acting granulocyte colony-stimulating factor administration, confirming that proactive myelosuppression prophylaxis greatly improves treatment safety and regimen completion rate. ③ Optimization of treatment sequence: Priority should be given to treating tumors with typical symptoms, high aggressiveness, or unclear diagnosis. The preoperative undetermined nature of the patient’s cervical lymph nodes rendered upfront thyroidectomy the optimal therapeutic choice in this clinical scenario: surgery realized radical control of PTC, meanwhile obtained pathological evidence to confirm DLBCL subtype, which prevented delayed anti-lymphoma therapy and reduced the progressive risk of both malignancies.

For this rare double-primary tumor, multidisciplinary collaboration covering surgery, hematology, pathology and radiology is the core guarantee for standardized diagnosis and management. Treatment should balance the pathophysiological characteristics, clinical staging of both tumors, and the patient’s individual tolerance, optimize the treatment sequence, and adopt an individualized comprehensive treatment regimen of radical surgery combined with immunochemotherapy. Combined with targeted adverse reaction management, this strategy achieved effective short-term tumor control and satisfactory safety outcomes in the present case. However, considering the limitation of only 6 months of follow-up, long-term survival benefit and sustained relapse-free efficacy cannot be conclusively determined. A standardized long-term follow-up protocol is therefore essential to dynamically monitor potential tumor recurrence and disease progression, so as to further verify the long-term efficacy and safety of this individualized therapeutic strategy. This case also provides clinical evidence supporting our proposed hypothesis: sustained overactivation of the NF-κB/PI3K/AKT signaling cascade and systemic immunosuppressive microenvironment are the core shared molecular foundations for the co-occurrence of PTC and ABC-type double-expressing DLBCL, which deserves further basic experimental validation.

## Data Availability

The datasets presented in this study can be found in online repositories. The names of the repository/repositories and accession number(s) can be found in the article/**Supplementary material**.
