# Supplementary material for: A case report and diagnostic-therapeutic analysis of de novo bilateral papillary thyroid carcinoma complicated with stage I a diffuse large B-cell lymphoma (ABC subtype, double-expressor)
Source: Front Oncol. 2026 Sep 21;16:1921091. doi: 10.3389/fonc.2026.1921091 (PMC13634846; doi:10.3389/fonc.2026.1921091)
Supplement: Supplementary file 1 [file Table1.docx]

**Supplementary Table 1. Baseline Laboratory Examination Results on Admission**

| **Project** | **Result** | **Range** |
| --- | --- | --- |
| WBC | 3.64 | 3.5-9.5*10^9^ /L |
| RBC | 3.86 | 3.8-5.1*10^12^ /L |
| Hb | 124 | 115-150 g/L |
| PLT | 176 | 125-350*10^9^ /L |
| ALT | 5 | 7-40 U/L |
| AST | 13 | 13-35 U/L |
| GGT | 10 | 7-45 U/L |
| ALP | 46 | 35-135 U/L |
| CREA | 55 | 41-73 umol/L |
| UREA | 3.1 | 2.6-7.5 mmol/l |
| UA | 198 | 155-391 umol/l |
| K | 4.3 | 3.5-5.3 mmol/l |
| Na | 142 | 137-147 mmol/l |
| Cl | 110 | 90-110 mmol/l |
