# Supplementary material for: A case report and diagnostic-therapeutic analysis of de novo bilateral papillary thyroid carcinoma complicated with stage I a diffuse large B-cell lymphoma (ABC subtype, double-expressor)
Source: Front Oncol. 2026 Sep 21;16:1921091. doi: 10.3389/fonc.2026.1921091 (PMC13634846; doi:10.3389/fonc.2026.1921091)
Supplement: Supplementary file 2 [file Table2.docx]

**Supplementary Table 2. Postoperative Baseline Laboratory Examination Results**

| **Project** | **Result** | **Range** |
| --- | --- | --- |
| WBC | 3.70 | 3.5-9.5*10^9^ /L |
| RBC | 3.48 | 3.8-5.1*10^12^ /L |
| Hb | 118 | 115-150 g/L |
| PLT | 189 | 125-350*10^9^ /L |
| ALT | 30 | 7-40 U/L |
| AST | 24 | 13-35 U/L |
| GGT | 21 | 7-45 U/L |
| ALP | 51 | 35-135 U/L |
| CREA | 52 | 41-73 umol/L |
| UREA | 6.1 | 2.6-7.5 mmol/l |
| UA | 225 | 155-391 umol/l |
| K | 4.5 | 3.5-5.3 mmol/l |
| Na | 139 | 137-147 mmol/l |
| Cl | 106 | 90-110 mmol/l |
