# Supplementary material for: A case report and diagnostic-therapeutic analysis of de novo bilateral papillary thyroid carcinoma complicated with stage I a diffuse large B-cell lymphoma (ABC subtype, double-expressor)
Source: Front Oncol. 2026 Sep 21;16:1921091. doi: 10.3389/fonc.2026.1921091 (PMC13634846; doi:10.3389/fonc.2026.1921091)
Supplement: Supplementary file 4 [file Table4.docx]

**Supplementary Table 4. Routine Blood Test Results During Follow-Up After Chemotherapy Completion**

| **Project** | **Result** | **Range** |
| --- | --- | --- |
| WBC | 7.16 | 3.5-9.5*10^9^ /L |
| NEUT | 5.68 | 1.8-6.3*10^9^ /L |
| RBC | 3.94 | 3.8-5.1*10^12^ /L |
| Hb | 125 | 115-150 g/L |
| PLT | 176 | 125-350*10^9^ /L |
